# Environmental Enrichment Attenuates Morphine-Induced Conditioned Place Preference and Locomotor Sensitization in Maternally Separated Rat Pups

**DOI:** 10.32598/bcn.9.4.241

**Published:** 2018-07-01

**Authors:** Soheyla Khalaji, Imanollah Bigdeli, Raheb Ghorbani, Hossein Miladi-Gorji

**Affiliations:** 1. Department of Psychology, Faculty of Psychology and Educational Sciences, Semnan University, Semnan, Iran.; 2. Department of Psychology, Faculty of Educational Sciences and Psychology, Ferdowsi University of Mashhad, Mashhad, Iran.; 3. Social Determinates of Health Research Center, Semnan University of Medical Sciences, Semnan, Iran.; 4. Laboratory of Animal Addiction Models, Physiology Research Center, Semnan University of Medical Sciences, Semnan, Iran.; 5. Department of Physiology, School of Medicine, Semnan University of Medical Sciences, Semnan, Iran.

**Keywords:** Maternal separation, Enriched environment, Conditioned place preference, Morphine, Behavioral sensitization

## Abstract

**Introduction:**

This study investigated the effect of the environmental enrichment during adolescence on morphine-induced Conditioned Place Preference (CPP) and locomotor sensitization in maternally separated male and female rat pups.

**Methods:**

Male Wistar rats were allowed to mate with female virgin Wistar rats. Pups were separated from them 3 hours per day during 2–14 days postnatal. All pups were weaned at 21 Postnatal Day (PND) and reared in standard environment or enriched environment from 21 to 50 PND with litter-mates of the same sex. The CPP and behavioral sensitization to morphine were assessed by an unbiased place conditioning paradigm and open filed method.

**Results:**

The results showed that the maternal separation enhanced morphine-induced CPP in both sexes, locomotor sensitization in male pups and tolerance to morphine-induced motor activity in female pups during adolescence. While, male and female pups reared in an EE exhibited a decrease in morphine-induced CPP, locomotor sensitization and tolerance induced by maternal separation compared to their control pups.

**Conclusion:**

Access to enriched environment during adolescence may have a protective effect against morphine-induced reward, locomotor sensitization and tolerance in adolescent male and female rats following maternal separation.

## Highlights

- Maternal separation increases morphine preference and locomotor sensitization.
- Environmental enrichment decreases conditioned place preference in maternally-separated pups.
- Environmental enrichment attenuates locomotor sensitization in maternally-separated pups.

## Plain Language Summary

According to human studies, it is unclear whether the adverse outcomes of childhood experiences have actually been established and then reversed. In this regard, preclinical studies of early stress in rodents may help.

## 1. Introduction

Previous studies have shown that exposure to negative early life events such as maternal separation increases the vulnerability to behavioral and physiological deficits in adulthood (Champagne, Francis, Mar, & Meaney, 2003) and disruption of the enkephalinergic system (Vazquez et al., 2005). Also, it has been reported that maternal separation increases vulnerability to substance use (Dube, Cook, & Edwards, 2010; Sinha, 2008), amphetamine (Campbell & Spear, 1999), morphine-induced Conditioned Place Preference (CPP) (Vazquez, Weiss, Giros, Martres, & Daugé, 2007), and morphine-induced sensitization and tolerance (Vazquez et al., 2005).

It seems that the Environmental Enrichment (EE) models can prevent functional brain alterations due to maternal separation. Our previous findings suggest that EE is probably a useful method for the prevention of the brain alterations induced by maternal separation. The EE contains physical stimuli, running wheel, rubber balls, tunnels, toys which stimulate exploration behavior in laboratory animals (Hajheidari, Miladi-Gorji, & Bigdeli, 2015a; Simpson & Kelly, 2011). We have previously shown that EE reduced the severity of drug dependence, voluntary consumption of drug, and anxiety/depressive-like behavior in morphine (Hammami-Abrandabadi, Miladi-Gorji, & Bigdeli, 2016) and methamphetamine withdrawn rats (Hajheidari et al., 2015a; Hajheidari, Miladi-Gorji, & Bigdeli, 2015b).

Some evidence indicates that EE reduces expression of morphine-induced CPP in male C57BL/6 mice (Xu, Hou, Gao, He, & Zhang, 2007), vulnerability to cocaine addiction (Nader et al., 2012), and sensitization to morphine (Bardo, Robinet, & Hammer Jr, 1997; Xu et al., 2007), cocaine (Solinas, Chauvet, Thiriet, Rawas, & Jaber, 2008) and stress reactivity induced by maternal separation (Francis, Diorio, Plotsky, & Meaney, 2002). Thus, the present study aimed to investigate whether exposure to EE during adolescence can attenuate morphine-induced CPP and locomotor sensitization in maternally separated male and female rat pups.

## 2. Methods

### 2.1. Animals

Male Wistar rats (250±10 g) were allowed to mate with female virgin Wistar rats (250±10 g) (n=14) during a 24 h period as described previously in our laboratory (Akhavan et al., 2013; Haydari, Miladi-Gorji, Mokhtari, & Safari, 2014) with a 12:12 h light/dark cycle, at temperature 22±4°C and food and water ad libitum throughout the experiment. Post-natal 0 (PND0) was the day of birth and maternal separation was conducted from PND2 to PND14 for 180 min. The pups were carried to an adjacent room with their original cage while room temperature was maintained at 32±0.5°C (days 2–5) or 30±0.5°C (days 6–14) for 3 h (Taghavi-Khalil Abad, Miladi-Gorji, & Bigdeli, 2016; Francis et al., 2002; Geuzaine & Tirelli, 2014). Control pups were reared under standard conditions. All pups were weaned on PND21 and housed with litter-mates of the same sex.

**Figure 1.**
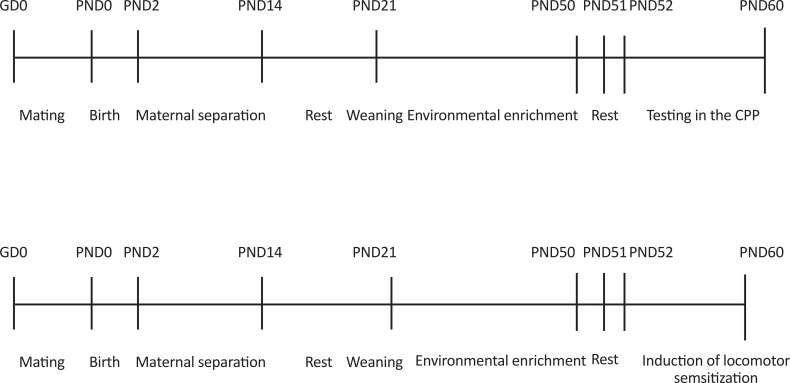
Timeline of experiments Maternal separation was conducted from PND2 to PND14. All pups were weaned on PND21 and housed in a standard environment or environmental enrichment. The pups were divided into two separate experiences (Experiment 1: Morphine-induced CPP and Experiment 2: Morphine-induced locomotor sensitization). All pups were tested with regard to the CPP and the open field chamber from PND52 to PND60. Abbreviations: PND: Postnatal Day; GD: Gestational Day

To decrease litter-size-induced variability in body weight of pups and to homogenize possible effects of genetic and prenatal factors, one or two pups of each sex from each litter were randomly assigned to each group. The pups (n=6–8/sex/experiment/rearing group) were randomly divided into four groups according to sex undergoing two separate experiments: Pups that were not separated from mothers and housed in a Standard Environment (SE) (No MS/SE), pups that were not separated from mothers and housed in an EE (No MS/EE); pups separated from mothers and housed in a SE (MS/SE), and pups separated from mothers and housed in an EE (MS/EE). Pups were reared in EE or SE for 4 weeks from PND21 to PND50. Then, all pups were put in standard cages on PND51. All animals were tested with regard to the CPP and the open field chamber from PND52 to PND60 (Figure 1).

### 2.2. Enriched environmental conditions

The EE consisted of large cages (96×49×38 cm) containing plastic tunnels, rope, swing, balls, ramp, ladder, shelters, step, cube and a running wheel, which were cleaned and changed every 2–3 days to maintain its novelty, with food and water ad libitum (Hajheidari et al., 2015a). The control animals were placed in standard cages as the SE condition. The SE consisted of standard plastic cage (42×34×15 cm). The pups were housed 6–9 per cages in both of EE and SE housing.

### 2.3. Assessment of conditioned place preference

The place preference apparatus was made of wood and consisted of two distinct compartments A and B (30×30×30 cm) with a black background or white and different pattern of white or black stripes (vertical or horizontal). It is separated from each other by a neutral area (30×15×30 cm) with a red background and having guillotine gates. This study was performed for 9 days as described previously (Taghavi-Khalil Abad et al., 2016; Xu et al., 2007), and the animals’ behavioral activity (time spent and the number of visits to the each camber with back-and-forth motions during pre- and post-conditioning test sessions, to determine that locomotor activity was not different between groups) was recorded by a video camera, using a tracking system (EthoVision, Noldus, The Netherlands).

#### 2.3.1. Pre-conditioning

On day 1 (Habituation), rats were placed on the neutral area and given free access to the entire chamber for 20 min in order to adapt to the environment. This test was repeated on day 2; the time spent in each of the three compartments was recorded and the initial preference was calculated. If the animal spent more than 60% of the time on day 2 in either side (initial side preference) it was eliminated from the experiment.

#### 2.3.2. Conditioning

Rats were treated with morphine 5 mg/kg, s.c. on days 3, 5, and 7 and saline on days 4, 6, and 8 during the conditioning phase. Drug and saline administrationalternated daily such that, half of the rat received drug in the chamber A and the other half received drug in the chamber B. Then each rat was placed into one of the two choice compartments for 15 min.

#### 2.3.3. Post-conditioning

On day 9, rats were placed on the center compartment and allowed to explore the entire chamber for 20 min. Preference or conditioning score is calculated by subtracting the amount of time spent in the drug-paired chamber before conditioning (on day 2) from the amount of time spent after conditioning.

### 2.4. Assessment of locomotor sensitization

Morphine-induced locomotor sensitization was assessed by the open field chamber. It consistes of a clear glass cylinder 25 cm in diameter and 30 cm high on a wooden plate with the same diameter. It was divided into 4 equal zones by two intersecting lines as described previously (Taghavi-Khalil Abad et al., 2016; Sahraei et al., 2006). First, baseline locomotor activity was evaluated. Then, to induce locomotor sensitization, morphine (5 mg/kg) was subcutaneously injected once daily for 3 consecutive days. However, the acute locomotor response of morphine was then evaluated only after the first (acute) injection of morphine. Then, the rats did not receive any treatment for the next 5 days.

All rats were challenged with morphine (1 mg/kg, s.c.) on the sixth day after a 5-day drug-free period, and evaluated for locomotor activity (post-morphine challenge), so that rats were placed on the open field chamber and allowed to explore the chamber for 10 min in order to adapt to novel environments. Then, the number of lines crossed for each rat with all four legs were counted manually for the next 10 min. After each test the arena was cleaned with 90% alcohol solution.

### 2.5. Statistical analysis

The obtained data are expressed as the mean±Standard Error of the Mean (SEM). The data were analyzed by using three-way analyses of variance (ANOVA) with the fixed factors of maternal care (No MS and MS), housing condition (SE and EE) and sex (male and female). Post hoc analysis included Tukey test. Statistical differences were considered significant at P<0.05.

## 3. Results

### 3.1. Decrease in morphine-induced CPP in maternally separated pups due to environmental enrichment

**Figure 2.**
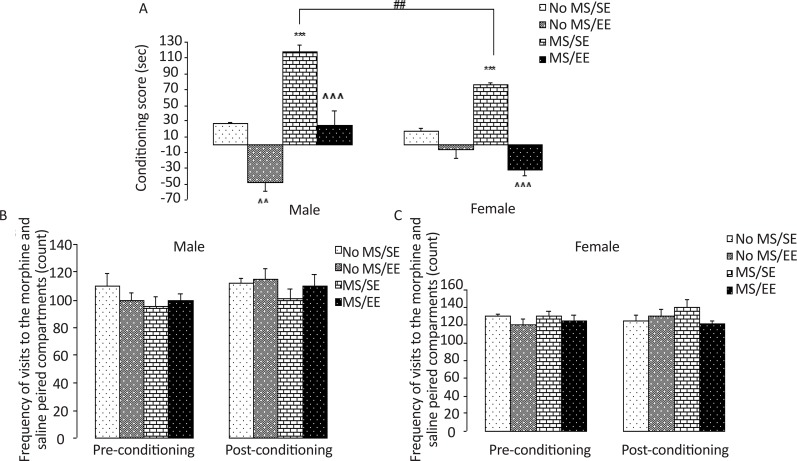
Effect of environmental enrichment on morphine-induced CPP in adult male and female rats following maternal separation (A) Morphine-induced place preference. The number of visits to the conditioning compartments in male (B) and female (C) rats. In this experiment, morphine-induced CPP was assessed by the place preference apparatus in maternally separated male and female rats. Preference score was lower in the No MS/EE male group and higher in the male and female MS/SE groups compared to those in No MS/SE group, while preference score was lower in MS/EE group than MS/SE group in both sexes. There was no significant difference in locomotor activity between both sexes of rats on the pre-conditioning and post-conditioning days. The obtained data were expressed as mean±SEM. ^^P=0.003; *** P=0.0001 vs. No MS/SE; ^^^P=0.0001 vs. MS/SE; ##P=0.003 vs. MS/SE male rats Abbreviations: No MS/SE: Not Separated from Mothers and housed in a Standard Environment; No MS/EE: Not Separated from Mothers and housed in an Environmental Enrichment; MS/SE: Separated from mothers and housed in a SE; MS/EE: Separated from Mothers and housed in an EE.

The results of the CPP are illustrated in Figure 2A. The analysis revealed a significant effect of maternal care (F_1, 47_=52.16, P=0.0001), and housing (F_1, 47_=121.49, P=0.0001), sex (F_1, 47_=6.01, P=0.018), maternal care×sex interactions (F_1, 47_=23.4, P=0.0001), maternal care×housing interactions (F_1, 47_=14.17, P=0.0001), and maternal care×housing×sex interactions (F_1, 47_=5.95, P=0.018). Between groups comparisons showed that the morphine-induced place preference score was lower in the No MS/EE male group (P=0.003) and higher in the MS/SE group compared to that in the No MS/SE group in male and female rats (both, P=0.0001).

While the place preference score for the morphine-paired compartment in the MS/EE group was significantly less than the MS/SE group in male and female rats (both, P=0.0001). Also, preference score was higher in MS/SE male rats than female (P=0.003). In summary, our study demonstrates that maternal separation enhanced morphine-induced CPP and EE attenuates the rewarding effect of morphine in both sexes compared to their control pups.

Also, EE severely attenuated morphine-induced CPP in the No MS/EE male group compared to the No MS/SE group. Moreover, there were no significant sex (F_1, 47_=1.23, NS), (F_1, 47_=2.3, NS), maternal care (F_1, 47_=0.28, NS), (F_1,47_=0.92, NS) and housing (F_1, 47_=3.13, NS), (F_1, 47_=0.75, NS) effects and interaction among them (F_1, 47_=1.32, NS), (F_1, 47_=1.95, NS) in the frequency of crossing from conditional and non-conditional part in pre-conditioning and post-conditioning situation, respectively. Thus, there was no difference in locomotor activity between groups in preconditioning and post-conditioning.

### 3.2. Decrease in morphine-induced locomotor sensitization in maternally separated pups due to environmental enrichment

**Figure 3.**
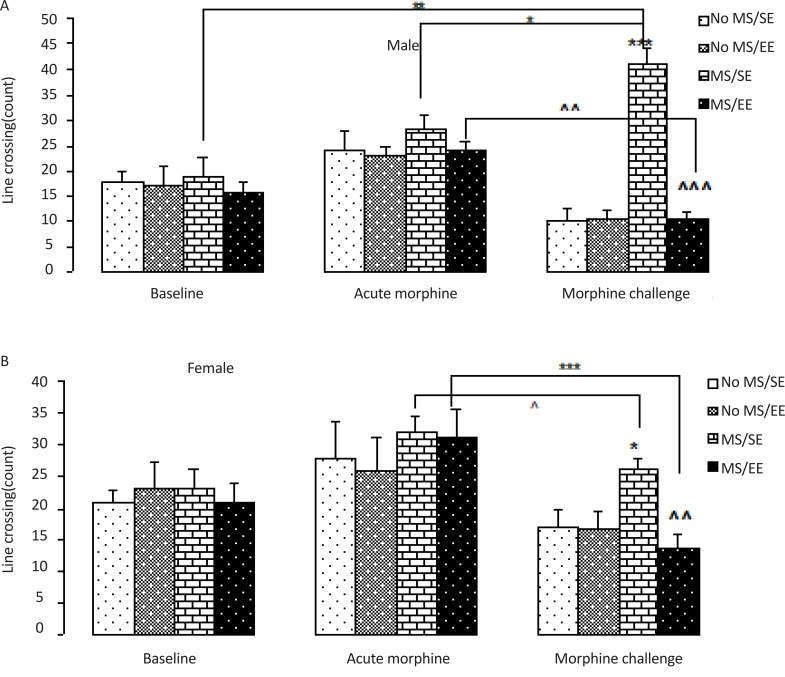
Effect of environmental enrichment on morphine-induced locomotor sensitization in male and female maternally separated rats In this experiment, morphine-induced locomotor sensitization was assessed by the open field chamber in maternally separated male and female rats. We found that the number of lines crossed was higher and lower in MS/SE male and female rats, respectively after morphine challenge; while it was lower in rats of both sexes in MS/EE groups. The obtained data were expressed as mean ±SEM. In A; *P=0.013; **P=0.007; and ***P=0.0001 vs. No MS/SE; ^^^ P=0.0001 vs. MS/SE; ^^ P=0.003 vs. acute morphine. In B; *P=0.049 vs. No MS/SE; ^^P=0.007 vs. MS/SE; *** P=0.004; ^P=0.03 vs. acute morphine Abbreviations: No MS/SE: Not Separated from Mothers and housed in a Standard Environment; No MS/EE; Not Separated from Mothers and housed in an Environmental Enrichment; MS/SE: Separated from Mothers and housed in a SE; MS/EE: Separated from Mothers and housed in an EE.

The results of the open field chamber using a three-way ANOVA are illustrated in Figure 3. There were no significant effect of sex, maternal care and housing as well as no interaction of maternal care×housing×sex in the number of lines crossed after baseline and the acute injection of morphine.

However, the analysis of morphine challenge-induced locomotor activity revealed a significant effect of maternal care (F_1, 52_=27.2, P=0.0001), and housing (F_1, 52_=35.4, P=0.0001) but no significant effects of sex (F_1, 52_=0.32, NS), significant maternal care×housing interactions (F_1, 52_=34.9, P=0.0001), maternal care×sex interactions (F_1, 52_=11.55, P=0.001), housing×sex interactions (F_1, 52_=5.47, P=0.023) and maternal care×housing×sex interactions (F_1, 52_=6.35, P=0.015). Comparisons between groups showed that the number of lines crossed in the MS/SE group was significantly higher after morphine challenge than that in No MS/SE group in male (P=0.0001) and female (P=0.049) pup rats. While the number of lines crossed in the MS/EE group was less than the MS/SE groups in male (P=0.0001) and female rats (P=0.007) after morphine challenge.

Also, between-group comparisons showed that the number of lines crossed was higher in MS/SE male rats after morphine challenge compared to the baseline response and the acute response of morphine (P=0.007, P=0.013; respectively), while was lower in MS/EE male rats after morphine challenge compared to the acute response of morphine (P=0.003). The number of lines crossed was lower in MS/SE and MS/EE female rats after morphine challenge compared to the acuteresponse of morphine (P=0.004, P=0.03).

In summary, our study demonstrates that maternal separation enhances locomotor sensitization in male pups and tolerance to morphine-induced motor activity in female pups during adolescence and EE decreases loco-motor sensitization and morphine tolerance compared to their control pups (MS/SE).

## 4. Discussion

We found that the maternal separation in rats would enhance morphine-induced CPP in both sexes, which was more evident in male pups than female ones; probably due to high basal levels of corticosterone in female rats (Rubio et al., 1998) in response to stress. Our finding is consistent with previous studies showing that the maternal separation enhances morphine-induced CPP (Taghavi-Khalil Abad et al., 2016; Vazquez et al., 2007) and cocaine self-administration (Moffett et al., 2006). It may be due to lower dopamine transporter expression (Brake, Zhang, Diorio, Meaney, & Gratton, 2004; Meaney, Brake, & Gratton, 2002), GABAA, receptor levels (Caldji, Francis, Sharma, Plotsky, & Meaney, 2000) and serotonin reuptake transporter expression (Lee et al., 2007), oxytocin (Amini-Khoei et al., 2017) and BDNF levels (Roceri, Hendriks, Racagni, Ellenbroek, & Riva, 2002) following maternal separation. This study provides novel evidence that exposure to EE for 30 days after maternal separation decreases morphine-induced CPP in male and female pups during adolescence. Although, previous studies have shown that EE reduces morphine-induced CPP (Xu et al., 2007) and self-administration of cocaine and amphetamine (Gipson, Beckmann, El-Maraghi, Marusich, & Bardo, 2011; Green, Gehrke, & Bardo, 2002). Given that maternal separation plays a major role in dysregulation of reward function (Ploj, Roman, & Nylander, 2003a; Ploj, Roman, & Nylander, 2003b), EE can activate the same pathways that are activated by morphine and may also lead to neuroplastic changes in the mesolimbic reward pathway (Thiel, Pentkowski, Peartree, Painter, & Neisewander, 2010).

Also, it may be due to increase in brain serotonin levels following EE (Koh, Magid, Chung, Stine, & Wilson, 2007). Future studies should examine the underlying neurobiological mechanisms. Based on our findings, there was no significant difference between the male and female rats in the number of visits to the conditional and unconditional chambers during pre-conditioning and post-conditioning phase. Thus, a higher preference score for morphine in the maternally separated rats was not directly due to an increase in the number of visits to the chambers.

We also found that the EE decreased morphine-induced CPP in the No MS male rats. Thus, the observed effects of EE in the MS male group did not merely nullify the impact of maternal separation. This finding indicates that rearing of No MS male rats in an EE before the onset of the CPP conditioning has made morphine less effective to induce a CPP. It implies that repeated activation of morphine reward system by EE decreased the rewarding effect of morphine, possibly through functional changes in mesolimbic dopamine transmission. In this regard, we have previously shown that exposure to EE partially decreases the incentive motivation for morphine intake, which can reduce the risk of sensitivity and drug seeking after withdrawal (Hammami-Abrandabadi et al., 2016). Therefore, this is not to say that might occur aversion in maternally separated pups. For example, it was shown that cocaine concomitantly activates neural circuits producing both reward and aversive behaviors (Kim, Pollak, Hjelmstad, & Fields, 2004).

Also, it has shown that the neural circuits involved in drug reward are distinct from those involved in drug aversion (Bardo & Bevins, 2000). Thus, the expression of both CPP and Conditioned Place Aversion (CPA) reflect drug-experience dependent plasticity (Kim et al., 2004). Contrary to previous work (Brielmaier, Mc-Donald, & Smith, 2012), negative CPP score should not necessarily indicate aversion. Also previous studies (Bardo & Bevins, 2000; Lima et al., 2017) have suggested that both CPP and CPA have distinct mechanisms. Future studies should examine whether exposure to EE can produce CPA or not.

We also found for the first time that morphine challenge-induced locomotor activity was higher and lower in the male and female MS/SE pups compared to the acute response of morphine which was reduced by EE in both sexes. Similar to previous studies, these findings indicate that maternally separated male and female pups become sensitized and tolerant to repeated morphine treatments; respectively (Taghavi-Khalil Abad et al., 2016; Kalinichev, Easterling, & Holtzman, 2001; Kalinichev, Easterling, & Holtzman, 2002). It may be due to increase in anxiety-like behaviors and oversecretion of corticosterone (Kalinichev, Easterling, Plotsky, & Holtzman, 2002), activation of the HPA axis (Pihoker, Owens, Kuhn, Schanberg, & Nemeroff, 1993) during adolescence.

On the whole, these findings indicate that greater sensitivity to the reinforcing properties of morphine, modulation of neurotransmitter release, and stressor-induced corticosterone responses following maternal separation could contribute to the enhanced CPP and locomotor sensitization induced by morphine in both males and females pups during adolescence. These sex-related differences in morphine-induced reward and locomotor sensitization may reflect differences in the effects of maternal separation on stress-reactivity (Plotsky & Meaney, 1993), the faster onset of corticosterone secretion in female rats (Young, Altemus, Parkison, & Shastry, 2001), and sex differences in glucocorticoid receptor, dopamine transporter expression (Kikusui, Faccidomo, & Miczek, 2005); all point to further research in this topic.

Our results support that access to EE following maternal separation can decrease the morphine-induced CPP and prevents the development of locomotor sensitization or tolerance to morphine in male and female rat pups during adolescence. Thus, access to EE could be exploited in the development of new therapeutic approaches for drug abuse prevention following negative early life events including maternal separation.

## Ethical Considerations

### Compliance with ethical guidelines

All experimental procedures were conducted in accordance with the National Institutes of Health’s Guide for the Care and Use of Laboratory Animals.
